# Response: Commentary: Skilled Bimanual Training Drives Motor Cortex Plasticity in Children with Unilateral Cerebral Palsy

**DOI:** 10.3389/fnhum.2017.00619

**Published:** 2017-12-15

**Authors:** Ya-Ching Hung, Kathleen M. Friel, Andrew M. Gordon

**Affiliations:** ^1^Family, Nutrition, and Exercise Sciences, Queens College, City University of New York, Flushing, NY, United States; ^2^Neurology and Neuroscience, Burke Medical Research Institute, White Plains, NY, United States; ^3^Biobehavioral Sciences, Teachers College, Columbia University, New York, NY, United States

**Keywords:** pediatrics, rehabilitation, transcranial magnetic stimulation, kinematics, neuroplasticity, hemiplegia

We thank Dr. Serrien for her interest in our paper and for the opportunity to clarify the differences between using clinical measures and kinematic measures to assess bimanual proficiency changes in children with unilateral spastic cerebral palsy (USCP). The primary clinical measure in Friel et al. (2016), the Assisting Hand Assessment (AHA), quantifies the quality of spontaneous use of the more-affective hand during bimanual activities. The AHA is validated and represents “expected outcomes” to quantify meaningful changes in functional ability at the International Classification of Functioning, Disability and Health (ICF) activity level, which describes human functioning including both capacity and performance. We concur that measures that assess bimanual coordination patterns in relation to individual task components could provide additional insights about bimanual coordination pattern strengthening following training. Dr. Serrien has been at the forefront of quantifying bimanual coordination (e.g., Serrien, 2008). She used tasks such as a bimanual drawer-opening task, in which one hand opens a pull-drawer and the other hand manipulates its contents, that she adapted with Dr. Mario Wiesendanger (Serrien and Wiesendanger, 2001) from his non-human primate studies (Kazennikov et al., 1994). We have adapted this task to quantify bimanual coordination in children with USCP and demonstrated specific deficits in bimanual spatio-temporal control compared to typically-developing children (Hung et al., 2004, 2010). Children with USCP were found to have reduced movement overlap of the two hands (i.e., moved each hand more sequentially) and thus longer durations between completion of the goal for each hand (drawer-opening and content manipulation, respectively). In fact, this assessment was sensitive in determining differences between unimanual and bimanual intensive treatments (Hung et al., 2011) while clinical outcomes were not (Gordon et al., 2011). Importantly, in a subset of participants in the Friel et al. (2016) study, kinematics during the same bimanual drawer-opening task were in fact subsequently examined (Hung et al., 2017). While there were no group AHA differences between the structured skill and unstructured practice groups, kinematic differences in bimanual drawer-opening were observed. Specifically, children in the structured skill group showed improvements in trunk and elbow movement control after training. Such improvements in more affected upper extremity movement control may correspond with the increase in motor map size of the more affected hand (Friel et al., 2016). Interestingly, no group differences were found for temporal measurements in the bimanual coordination pattern (both groups improved), while there were differences between the two groups in motor map changes (i.e., the structured skill group had expansion of the M1 motor map whereas the unstructured practice group did not).

Dr. Serrien correctly noted that the changes in cortical plasticity for the structured practice group were independent of the hemisphere of control, and the fact that both hemispheres respond to the bimanual training is in contrast with the findings of Kuhnke et al. (2008), who showed that functional gains are less pronounced in children with ipsilateral than contralateral control of the affected hand following unimanual training (constraint-induced movement therapy). While we agree that it is essential that rehabilitation approaches are optimized across the pathology of USCP, we should point out that we have shown that children with ipsilateral and children with contralateral control of the affected hand show similar levels of improvement following bimanual training (Smorenburg et al., 2017). One could argue that the current TMS analysis examined only the more affected hand, rather than areas associated with bimanual movement (Serrien, 2017). Friel et al. (2016) suggested that plasticity may have occurred in other brain regions. Recently we showed that structure (using diffusion tensor imaging) and function (using electroencephalography) of the somatosensory system is also highly related to the magnitude of a child's hand movement impairment (Gupta et al., 2017). Although we do not yet know how the somatosensory system may change after intensive hand therapy, the tight association between sensory system function and hand impairment suggests that sensory system plasticity may also underlie the improvements produced by intensive therapy. Assessing plasticity in other brain regions, however, often requires the use of techniques that are expensive and difficult to use with children, such as functional magnetic resonance imaging (fMRI). The compliance required of study participants for the collection of robust fMRI data is often beyond the capability of a child.

There are limitations associated with kinematic analyses. First, although they may be more sensitive to changes in movement coordination and strategy than clinical measures, unlike for gait, few upper extremity tasks are standardized with normative data. Furthermore, the cost and personnel required for this type of data collection/analysis limits wide-spread use in clinical research. Finally, although such analyses provide insights into spatio-temporal coordination, it puts the goal of normalizing movement at the forefront. In contrast, motor learning approaches such as bimanual training are aimed at increasing function, even when that requires compensation rather than normalization of movement. Nonetheless, we whole heartedly agree with Dr. Serrien that detailing the properties of motor circuits, including motor and sensory systems, along with interhemispheric connectivity, will further enhance the evaluation of rehabilitation interventions for USCP, specifically related to bimanual coordination. Our current work is aimed precisely at these goals.

## Author contributions

Y-CH wrote the first draft of this commentary. KF and AG edited and contributed to the writing of the final draft.

### Conflict of interest statement

The authors declare that the research was conducted in the absence of any commercial or financial relationships that could be construed as a potential conflict of interest.
